# Altered glutamate level and its association with working memory among patients with treatment-resistant schizophrenia (TRS): a proton magnetic resonance spectroscopy study

**DOI:** 10.1017/S003329172100533X

**Published:** 2023-05

**Authors:** Li-Chung Huang, Shih-Hsien Lin, Huai-Hsuan Tseng, Kao Chin Chen, Muhammad Abdullah, Yen Kuang Yang

**Affiliations:** 1 Institute of Clinical Medicine, National Cheng Kung University, Tainan, Taiwan; 2 Department of Psychiatry, Chia-Yi Branch, Taichung Veteran General Hospital, Chia-Yi, Taiwan; 3 Department of Counseling, National Chia-Yi University, Chia-Yi, Taiwan; 4 Department of Psychiatry, National Cheng Kung University Hospital, College of Medicine, National Cheng Kung University, Tainan, Taiwan; 5 Institute of Behavioral Medicine, College of Medicine, National Cheng Kung University, Tainan, Taiwan; 6 Taiwan International Graduate Program in Interdisciplinary Neuroscience, National Cheng Kung University and Academia Sinica, Taipei, Taiwan; 7 Department of Psychiatry, Tainan Hospital, Ministry of Health and Welfare, Tainan, Taiwan

**Keywords:** Anterior cingulate cortex, cognition, glutamatergic neurometabolites, magnetic resonance spectroscopy, treatment-resistant schizophrenia, working memory

## Abstract

**Background:**

Treatment-resistant schizophrenia (TRS) and non-TRS may be associated with different dopaminergic and glutamatergic regulations. The concept of dysregulated glutamatergic concentrations in specific brain regions remains controversial. Herein, we aimed to assess (i) the distribution of the glutamatergic concentration in the brain, (ii) the association between working memory (WM) differences in TRS and non-TRS patients, and (iii) whether an alteration in the glutamate (Glu) level is associated with WM.

**Methods:**

The participants included 38 TRS patients, 35 non-TRS patients, and 19 healthy controls (HCs), all of whom underwent 1.5-Tesla proton magnetic resonance spectroscopy of anterior cingulate cortex (ACC) and medial prefrontal cortex (MPFC). The ratios of glutamatergic neurometabolites to *N*-acetylaspartate + *N*-acetyl aspartylglutamate (NAAx) were calculated. Cognitive function was assessed using the Wechsler Adult Intelligence Scales, 4th Edition, which included the working memory index (WMI).

**Result:**

The TRS patients had a higher glutamate + glutamine (Glx)/NAAx ratio compared to the non-TRS patients and HCs in the ACC, but this was not significantly different in the MPFC. WM was negatively correlated with Glx/NAAx in the ACC among the non-TRS patients, but not in the TRS patients or HCs.

**Conclusions:**

Our findings were consistent with most studies indicating that the glutamatergic concentration in the ACC plays important roles in the classification of TRS and cognition. Our results may provide potential evidence for predictors and treatment response biomarkers in TRS patients. Further research is needed to probe the value using the relationship between Glu and WM as a potential prognostic predictor of schizophrenia.

## Introduction

Schizophrenia, a neurodegenerative disorder with functional decline, may become a burden on patients and their families, and results in increased medical costs and negative effects on public health (Charlson et al., 2018; McCutcheon, Marques, & Howes, 2020). Currently, one-third of patients with schizophrenia are defined as having treatment-resistant schizophrenia (TRS), and exhibit a poor medication response with current antipsychotic agents, including first- and second-generation antipsychotics, clozapine (as the last-line medication), and integrated antipsychotics with high dosages and appropriate treatment durations (Howes et al., 2017a). Based on the concepts of maternal infection, inflammation, genetic expression, stress on endocrine systems, and neurotransmitters, and in particular the dopaminergic and glutamatergic hypotheses, the mechanisms of schizophrenia and TRS are mutative (Howes, McCutcheon, Owen, & Murray, 2017b; Huang, Lin, Tseng, Chen, & Yang, 2020; Nucifora, Woznica, Lee, Cascella, & Sawa, 2019). Considering that first- and second-generation antipsychotic agents are mostly based on the dopaminergic hypothesis, glutamate neurometabolites might play another critical role in major psychiatric disorders, especially in schizophrenia and TRS (Li, Yang, & Lin, 2019). Some studies have indicated that dopamine activity in the striatum and imbalances in glutamatergic neurometabolites in all cortical regions of the brain may cause psychotic symptoms and functional decline (Howes, McCutcheon, & Stone, 2015; Huang et al., 2020; Lin, Hashimoto, & Lane, 2019a). Some efficacious medications based on the glutamatergic hypothesis, including *N*-methyl-d-aspartate (NMDA) receptor agonists, glycine transporter inhibitors, d-amino acid oxidase inhibitors, and anticonvulsants, have resulted in a good treatment response in patients with psychiatric disorders (Lin, Chen, & Lane, 2020a; Lin & Lane, 2019; Lin, Yang, Chen, Wang, & Lane, 2020b; Lin, Yang, & Lane, 2019b; Tzang, Chang, Chang, & Lane, 2019). Therefore, the integrated glutamatergic and dopaminergic hypotheses is considered one of the explanations for schizophrenia classification. The clinical implications of the integrated hypothesis may enable the additional use of glutamate as a predictor (Corcoran et al., 2020; Lin et al., 2019a; Tarumi et al., 2020) and biomarker of treatment response and cognitive function (Corcoran et al., 2020; Hutcheson et al., 2012; Kraguljac et al., 2018; Shah et al., 2020; Tarumi et al., 2020), respectively.

In *in vivo* studies, extracellular levels of glutamate neurometabolites, such as in the serum and cerebrospinal fluid, and intracellularly in various brain regions, have been measured (Borisova et al., 2018; Lin et al., 2019a). Several studies have shown that proton magnetic resonance spectroscopy (^1^H-MRS) is an effective method by which to estimate glutamate (Glu) and glutamine (Gln) concentrations in specific brain regions of patients with schizophrenia (Hutcheson et al., 2012; Shah et al., 2020; Tarumi et al., 2020; Wijtenburg et al., 2017). Unlike the dopaminergic system, which focuses on the substantia nigra and striatum (Huang et al., 2020; Tanaka, 2006), specific regions of interest (ROIs) for the measurement of glutamate activities in schizophrenia, including cortical areas such as the anterior cingulate cortex (ACC), dorsal lateral prefrontal cortex (DLPFC), hippocampus, thalamus, caudate, and medial prefrontal cortex (MPFC), are still being investigated (Conus et al., 2018; Corcoran et al., 2020; de la Fuente-Sandoval et al., 2018; Singh et al., 2018; Tarumi et al., 2020).

The ACC is located in a transport area of the brain, which connects the limbic system, controls emotion, and integrates the neuronal circuitry of affect, attention, dopaminergic activities and the prefrontal cortex, known to be associated with cognitive function (Stevens, Hurley, & Taber, 2011). In structural concepts, such as Brodman mapping or Vgot's system (Stevens et al., 2011), the ACC is divided into different subgroups anatomically, which may include the following: (1) the caudal or dorsal ACC or middle cingulate cortex; and (2) the ventral ACC, including pregenual regions and subgenual regions, which are considered part of the MPFC. Different brain regions present different patterns of glutamatergic neurometabolites in schizophrenia (Nakahara et al., 2021; Thomas, Bozaoglu, Rossell, & Gurvich, 2017). Most studies suggest that the ACC might be an important ROI, but the distribution of glutamate levels in this region may not be associated with the positive and negative syndrome scale (PANSS) severity, which is compatible with other evidence (Reddy-Thootkur, Kraguljac, & Lahti, 2020; Tarumi et al., 2020). Two of the most famous brain regions, the ACC and MPFC, have presented controversial results in terms of the glutamatergic levels in schizophrenia patients (Nakahara et al., 2021; Smucny, Carter, & Maddock, 2021; Thomas et al., 2017), though these two regions are anatomically adjacent in the brain. To date, the findings remain controversial, perhaps due to several important differences between studies. Specific brain regions in which the glutamate activity has been measured do not correspond with specific subgroups of schizophrenia, such as TRS (Shah et al., 2020; Tarumi et al., 2020), treatment-responsive or non-treatment-resistant schizophrenia (non-TRS) (Shah et al., 2020; Wijtenburg et al., 2017), first-episode psychosis (de la Fuente-Sandoval et al., 2018), ultra-TRS (Shah et al., 2020), and acute or chronic states (Reddy-Thootkur et al., 2020; Thomas et al., 2017; Wijtenburg et al., 2017). These studies do not consistently present a clear scheme of the glutamate hypothesis regarding neurometabolite fluctuations. In addition, the most appropriate scale of measurement, i.e. concentration or ratio, remains unclear.

Cognitive function decline is not only a measure of treatment outcome with medication or non-medication rehabilitation for schizophrenia, but also reduces a patient's quality of life, which may lead to a burden on their family (Fernandes, Cajão, Lopes, Jerónimo, & Barahona-Corrêa, 2018; Forbes, Carrick, McIntosh, & Lawrie, 2009; Guo, Ragland, & Carter, 2019). Cognitive deficits in schizophrenia have been categorized in the following domains (Guo et al., 2019): attention, working memory (WM), speed of processing, verbal and visual learning memory, and social cognition (Arnsten, Girgis, Gray, & Mailman, 2017; Bowie & Harvey, 2006; Fernandes et al., 2018). WM deficits in schizophrenia are represented by a decreased accuracy and an increased response time on measured tests, which have been observed in various subgroups of schizophrenia (Corcoran et al., 2020; Forbes et al., 2009; Guo et al., 2019), including early-onset (Mesholam-Gately, Giuliano, Goff, Faraone, & Seidman, 2009; White, Schmidt, & Karatekin, 2010), first-episode (Mesholam-Gately et al., 2009), and especially chronic schizophrenia patients (Mesholam-Gately et al., 2009). Though WM deficits are modulated by multiple brain regions, especially the ACC and MPFC (Dash, Moore, Kobori, & Runyan, 2007; Euston, Gruber, & McNaughton, 2012; Stevens et al., 2011), most studies have shown no significant association between WM and glutamate level, while some studies have presented divergent results. This may be due to the different brain regions examined, classifications of schizophrenia, and tools used for cognitive assessment (Reddy-Thootkur et al., 2020). WM has been classed as a crucial part of various test batteries, such as the Wechsler Adult Intelligence Scale-Fourth Edition (WAIS-IV) and the repeatable battery for the assessment of neuropsychological status (RBANS) in other studies (Reddy-Thootkur et al., 2020). Most neuroimaging findings suggest that brain regions such as the frontal-parietal network are affected in schizophrenia, which is the neuropathology that may influence WM impairment in schizophrenia patients (Deserno, Sterzer, Wüstenberg, Heinz, & Schlagenhauf, 2012). It has also been proposed that regarding the inverted U-shaped relationship between DLPFC activation and WM performance, in patients with schizophrenia, the relationship is shifted to the right side of the inverted U-shaped curve as compared with healthy controls (HCs) (Callicott et al., 2000; Cools & D'Esposito, 2011; Lenartowicz & McIntosh, 2005; Perlstein, Carter, Noll, & Cohen, 2001). Considering that both the ACC and MPFC are associated with WM in patients with schizophrenia, the glutamatergic distribution and mechanisms in these two closely situated brain regions related to cognition in patients with schizophrenia remain controversial (Reddy-Thootkur et al., 2020; Thomas et al., 2017). We speculated that the relationship between glutamate level and cognitive function could differ between subtypes of schizophrenia; more specifically, between TRS and non-TRS.

To address the effects of glutamatergic neurometabolites on cognitive function in the subgroups of schizophrenia, we hypothesized that the glutamatergic levels in specific brain regions, especially the ACC, differ among TRS, non-TRS, and HC groups, which may lead to differences in cognitive function. The aims of the current study were to (i) reconfirm that glutamatergic levels in TRS patients are higher than those in other subgroups, especially in the ACC; (ii) examine whether TRS and non-TRS patients exhibited poorer performances compared to HCs on the working memory index (WMI) of the WAIS-IV; and (iii) determine whether the associations between the WMI and glutamatergic levels differed between non-TRS and TRS patients.

## Methods

### Ethics statement

This cross-sectional ^1^H-MRS study was conducted at Taichung Veterans General Hospital, Chia Yi Branch, between 2018 and 2020. The study was approved by the ethics committees of a local medical center (IRB nos. A-ER-107-045 and B-ER-107-042), and the protocol was consistent with the principles outlined in an internationally recognized standard for ethical conduct in human research. All participants gave informed consent before enrolment in this study.

### Participants

This study included participants aged 20–55 years with a diagnosis of schizophrenia or schizoaffective disorder according to the Diagnostic Systematic Manual-5th edition. The participants were recruited from outpatient psychiatry clinics and a chronic psychiatric ward in the psychiatry department of Taichung Veterans General Hospital, Chia Yi Branch. Patients who had received no antipsychotic adjustment for at least 6 months prior to recruitment were enrolled in the study. TRS was defined as follows (Howes et al., 2017a): (i) no response to at least two non-clozapine antipsychotic trials, each administered at a dose equivalent to ⩾600 mg/day of chlorpromazine (CPZ) for ⩾6 weeks; (ii) a Clinical Global Impression-Severity (CGI-S) (Guy, 1976) score ⩾4; and (iii) at least two items of the positive symptoms of the PANSS scored ⩾4. Patients who did not meet the aforementioned criteria were classified as having non-TRS. The exclusion criteria were as follows: (i) abnormal brain structure disease, (ii) substance-related usage disorder, except nicotine usage, and (iii) severe medical diseases or pregnancy. HCs without psychiatric or organic mental disorders were recruited from the community and provided informed consent.

### Image parameters

All participants were scanned using a 1.5-T GE Sigma scanner with an eight-channel head coil. MRS was employed to assess the excitatory glutamate–glutamine (Glx) concentration. A three-dimensional *T*1-weighted inversion recovery magnetic resonance imaging (MRI) scan was performed on all subjects [axial MRI 3D brain volume, echo time (TE) = 2.9 ms, repetition time (TR) = 7.7, inversion time = 450 ms, flip angle = 12°, field of view = 230 mm, matrix size = 256 × 256, and slice thickness = 1.0 mm].

**Fig. 1.**
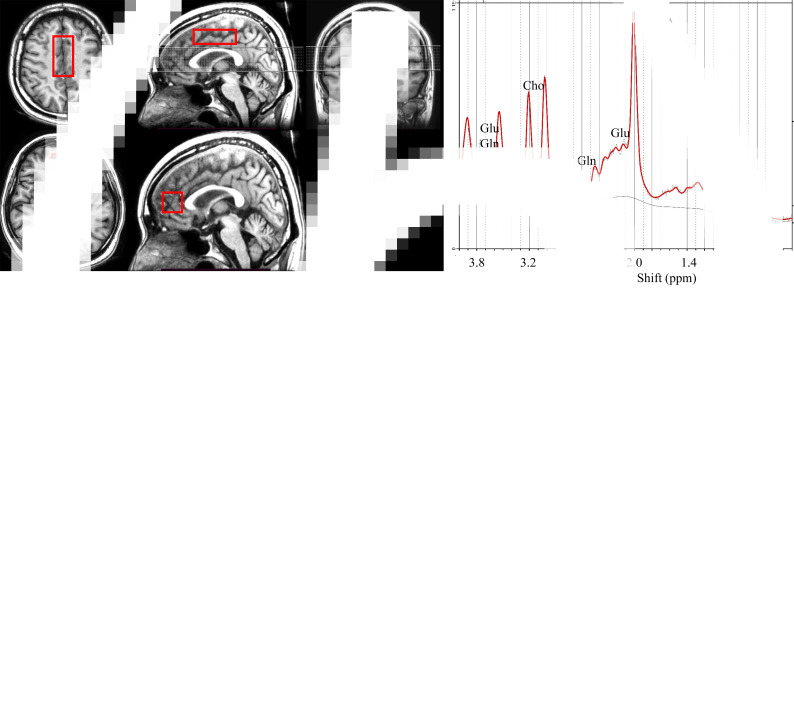
Voxel locations and placement procedures for ^1^H-MRS. ^1^H-MRS: proton magnetic resonance spectroscopy.

^1^H-MRS was performed using point-resolved spectroscopy (TE = 35 ms, TR = 2000 ms, spectral width = 2500 Hz, 2048 data points, 128 water-suppressed, 16 water-unsuppressed averages, and eight excitation numbers). The voxels were placed in the bilateral ACC (voxel size = 10.0 mL) and MPFC (voxel size = 10.0 mL), scanned for 9:20 (Ende et al., 2016; Milak et al., 2016). The region and voxel size of the ACC in the current study were similar to the dorsal ACC mentioned in previous articles (Stevens et al., 2011; Tarumi et al., 2020); the MPFC in this study included the partial pregenual ACC in its structure (Stevens et al., 2011). The detailed voxel placement procedures, locations of ^1^H-MRS voxels, and representative spectra are shown in Fig. 1. Spectra were preprocessed using the FID-Appliance (https://github.com/CIC-methods/FID-A), and concentrations of neurometabolites were measured using LC models. Signal-to-noise ratios ⩽10, full-width at half-maximum ⩾10 Hz, or standard deviation (s.d.) values ⩾20% were defined as being of poor quality and were excluded from the analyses. In this study, glutamate + glutamine (Glx) was the main neurometabolite outcome. MRS data were fitted and analyzed using LCModel (version 6.3-1K). Data on supplementary neurometabolites, including glutamate, glutamine, myoinositol, glycerophosphocholine + phosphocholine, *N*-acetylaspartate + *N*-acetyl aspartylglutamate (NAAx), and creatine + phosphocreatine, were also obtained.

### Clinical assessments

Clinical assessments included the following: PANSS score (Leucht et al., 2005), CGI-S score, Global Assessment of Functioning symptom severity score, and, specifically to assess WM, the WMI of the WAIS-IV (Benson, Hulac, & Kranzler, 2010).

### Statistical analyses

The demographic and clinical characteristics of the groups of participants were compared using Fisher's exact test for dichotomous variables. Differences between groups were tested using one-way analysis of variance (ANOVA), with the exception of differences between the non-TRS and TRS groups, for which the *t* test was used. Post-hoc pairwise comparisons were conducted with Bonferroni corrections for multiple comparisons. Similarly, one-way ANOVA was used to test the hypothesis of group differences in glutamatergic levels. Supplementary analysis of covariance (ANCOVA) was employed to test the robustness of the main finding after controlling some demographic factors, including sex, age, smoking status, educational level, and the use of certain drugs. To test the hypothesis of an association between WM and Glx/NAAx, Pearson correlation was used as the first tool, and Spearman's correlation analysis was employed for conclusive testing of an association owing to the distribution of the data. The threshold for statistical significance in all analyses was set at *p* < 0.05, with further correction for multiple comparisons when noted (for testing of correlations in the three groups, the control threshold was 0.0167). All statistical analyses were conducted using IBM SPSS Statistics, version 22 (IBM Corporation, Armonk, NY, USA).

## Results

The demographic and clinical characteristics, including smoking status, of the participants are shown in Table 1. There were no sex differences among the three subgroups. General tendencies toward an older age, a lower educational level, poorer clinical outcomes, and poorer WM were noted among the patient groups, especially the TRS patients. Other results, including scores on the WAIS-IV in domains except WM, and details of the results of treatment with clozapine, clozapine-like agents, first- and second-generation antipsychotics, lithium and mood stabilizers, are presented in online Supplementary Tables S1 and S2. Regarding the concentrations of neurometabolites (Table 2), higher Glx/NAAx in the ACC area was found in the TRS group in comparison with the non-TRS group; this difference remained even after adjustment for covariates (model 1 corrected for age and sex; model 2 corrected for age, sex, smoking status, educational level, and IQ). In addition, we examined whether the difference between the TRS and non-TRS groups remained after controlling for potential confounders, including age, sex, educational level, smoking status, and IQ. The difference remained significant. ANCOVA tables presenting data on neurometabolite levels in the ACC in the TRS and non-TRS patients are presented in online Supplementary Tables S3 and S4. There were no significant differences in neurometabolite levels in the MPFC between groups (as shown in online Supplementary Tables S5 and S6). The group difference in Glx in the ACC remained significant after controlling for the use of several drugs (mood stabilizers: online Supplementary Table S7; antipsychotic agents: online Supplementary Table S8; clozapine and clozapine-like agents: online Supplementary Table S9).

**Table 1.** Demographic data and clinical characteristics of the study participants, stratified into three groups

Characteristic	Non-TRS (*n* = 35)	TRS (*n* = 38)	Control (*n* = 19)	Test statistics		
	Mean (s.d.)	Mean (s.d.)	Mean (s.d.)	χ^2^/*t*/*F*	*p*	Post-hoc
Gender (male/female)	17/18	21/17	9/10	0.47	0.80	
Age (years)	39.77 (8.19)	43.58 (8.76)	38.05 (8.07)	3.28	0.04	TRS > non-TRS > control
Smoking status	16/19	13/25	2/17	6.83	0.03	Non-TRS > TRS > control
Education (years)	12.89 (2.37)	11.11 (2.45)	17.11 (1.91)	42.47	<0.01	Control > non-TRS > TRS
Duration of illness (years)	16.91 (7.56)	23.21 (8.37)		3.36	<0.01	
Dosage (CPZ equivalent)	467.91 (186.48)	821.36 (181.71)		8.2	<0.01	
Psychiatric ratings: PANSS						
Total score	38.23 (5.77)	83.21 (25.40)		10.23	<0.01	
Positive score	8.20 (1.35)	22.11 (6.02)		13.35	<0.01	
Negative score	7.69 (1.73)	17.47 (6.17)		9.06	<0.01	
General score	18.86 (3.56)	37.76 (14.75)		7.77	<0.01	
Aggression score	3.40 (0.98)	4.87 (3.10)		2.68	<0.01	
Cognitive function rating						
WMI	84.49 (15.01)	74.48 (14.23)	101.63 (16.13)	21.07	<0.01	Control > non-TRS > TRS

TRS, treatment-resistant schizophrenia; CPZ, chlorpromazine; PANSS, positive and negative syndrome scale; WMI, working memory index.

**Table 2.** Group comparison of the concentrations of neurometabolite covariates in the ACC and MPFC

(a) ACC						
	Non-TRS (*n* = 35)	TRS (*n* = 38)	Control (*n* = 19)	Test statistics		
	Mean (s.d.)	Mean (s.d.)	Mean (s.d.)	*F*	*p*	Post-hoc
Uncorrected model						
Glx/NAAx	1.081 (0.23)	1.27 (0.39)	1.13 (0.19)	4.06	0.021	Non-TRS < TRS
Corrected model 1						
Glx/NAAx (estimated mean, s.e.)	1.07 (0.04)	1.29 (0.05)	1.11 (0.07)	5.17	0.008	Non-TRS < TRS
Corrected model 2						
Glx/NAAx (estimated mean, s.e.)	1.06 (0.05)	1.25 (0.06)	1.20 (0.10)	3.60	0.032	Non-TRS < TRS

(b) MPFC						
	Non-TRS (*n* = 28)	TRS (*n* = 32)	Control (*n* = 15)	Test statistics		
	Mean (s.d.)	Mean (s.d.)	Mean (s.d.)	*F*	*p*	Post-hoc
Uncorrected model						
Glx/NAAx	1.49 (0.36)	1.59 (0.79)	1.44 (0.37)	0.38	0.69	
Corrected model 1						
Glx/NAAx (estimated mean, s.e.)	1.50 (0.11)	1.58 (0.11)	1.46 (0.15)	0.21	0.81	
Corrected model 2						
Glx/NAAx (estimated mean, s.e.)	1.51 (0.11)	1.60 (0.14)	1.41 (0.23)	0.19	0.82	

ACC, anterior cingulate cortex; MPFC, medial prefrontal cortex; TRS, treatment-resistant schizophrenia; Glx, total glutamate + glutamine; NAAx, *N*-acetylaspartate + *N*-acetyl aspartylglutamate; s.e., standard error; FSIQ, full-scale IQ.

Corrected model 1: adjusted for age and sex; pairwise comparison with Bonferroni correction.

Corrected model 2: adjusted for age, sex, smoking status, educational level, and FSIQ; pairwise comparison with Bonferroni correction.

**Fig. 2.**
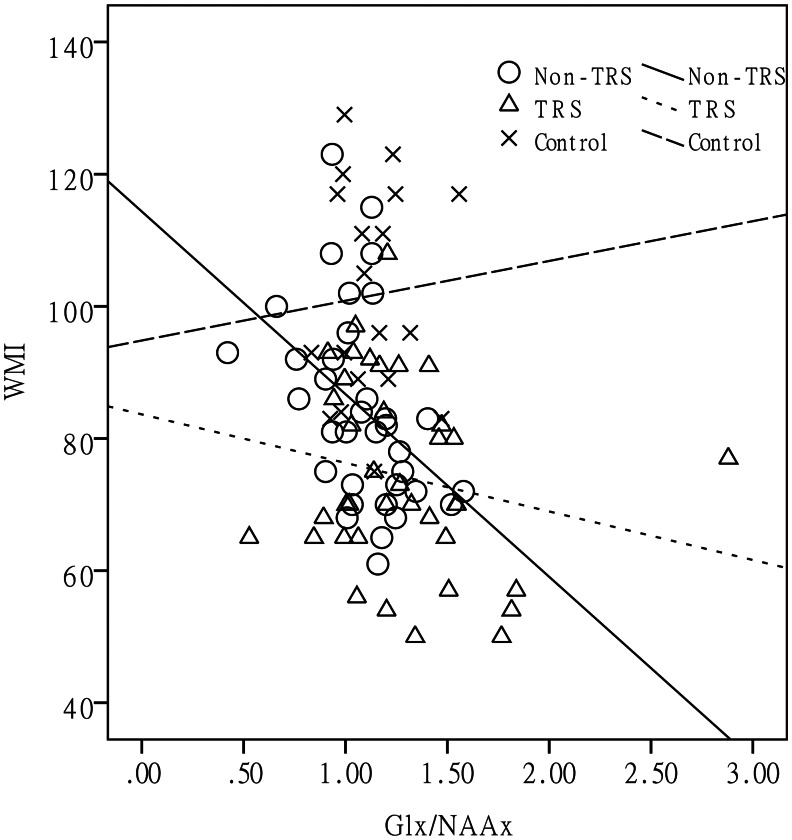
An association between the Glx/NAAx ratio in the ACC and WMI was observed (Spearman's *ρ* = −0.51, *p* = 0.002) in the non-TRS patients, but not in the TRS patients or HCs. TRS, treatment-resistant schizophrenia; Glx, total glutamate + glutamine; NAAx, *N*-acetylaspartate + *N*-acetyl aspartylglutamate; WMI, working memory index; ACC, anterior cingulate cortex.

Most clinical and demographic characteristics, including the total PANSS score, were not associated with neurometabolite levels in the groups (Table 3). To test this hypothesis, a series of stratified correlation analyses was conducted. The association between the Glx/NAAx ratio in the ACC and cognitive function (WMI) in the non-TRS patients was significant (Spearman's *ρ* = −0.51, *p* = 0.002, below the threshold after Bonferroni correction, *p* = 0.0167), while this association was not significant in the TRS patients and HCs (Fig. 2). Similar patterns were found for the Gln/NAAx ratio and WMI.

**Table 3.** Associations between demographic characteristics and the glutamatergic level in the ACC in the three groups

		Non-TRS (*n* = 35)		TRS (*n* = 38)		Control (*n* = 19)	
		Statistics	*p*	Statistics	*p*	Statistics	*p*
Sex	Glx/NAAx	0.75	0.46	1.50	0.14	0.14	0.89
	Glu/NAAx	0.23	0.82	1.77	0.08	0.39	0.70
Age	Glx/NAAx	−0.13	0.46	−0.24	0.15	0.37	0.12
	Glu/NAAx	−0.35	0.04*	−0.11	0.52	0.23	0.33
Educational level	Glx/NAAx	−0.36	0.84	−0.27	0.11	−0.09	0.73
	Glu/NAAx	0.15	0.41	−0.23	0.17	−0.18	0.06
WMI	Glx/NAAx	−0.42	0.01*	−0.2	0.23	0.07	0.78
	Glu/NAAx	−0.23	0.19	−0.15	0.38	0.36	0.14
PANSS	Glx/NAAx	−0.26	0.14	0.12	0.48	NA	
	Glu/NAAx	−0.16	0.54	0.09	0.61	NA	

TRS, treatment-resistant schizophrenia; ACC, anterior cingulate cortex; Glu, glutamate; Glx, total glutamate + glutamine; NAAx, *N*-acetylaspartate + *N*-acetyl aspartylglutamate; WMI, working memory index; PANSS, positive and negative syndrome scale.

*Note*: The statistics shown are from the *t* test for sex, while Pearson's *R* was used for the other demographic characteristics.

*p < 0.05.

## Discussion

The results of our study were in agreement with literature reporting that the ACC glutamatergic level in TRS patients is higher than in non-TRS patients and HCs (Goldstein, Anderson, Pillai, Kydd, & Russell, 2015; Mouchlianitis et al., 2016; Tarumi et al., 2020), similar to the integrated glutamatergic and dopaminergic hypothesis (Huang et al., 2020). In addition to the dopamine hypothesis, the ACC is one of the major areas that modulates positive and negative symptoms and cognitive function, via an underlying mechanism of abnormal distribution of glutamatergic levels with NMDA or α-amino-3-hydroxy-5-methyl-4-isoxazolepropionic acid receptor (AMPA) receptor dysfunction (Egerton et al., 2021; Gallinat, McMahon, Kühn, Schubert, & Schaefer, 2016). The phenomenon of neurotoxicity, such as psychotic symptoms caused by ketamine and phencyclidine usage, may also explain the role of the glutamatergic hypothesis in patients with schizophrenia. In contrast, the levels of glutamate neurometabolites were reduced in the MPFC in the schizophrenia patients relative to HC groups, while inconsistent results have been reported in the literature (Smucny et al., 2021). The reason for the non-positive findings in the MPFC is unclear; the glutamatergic concentration, assessed via MRS detection, may be affected by (1) susceptibility artifacts related to the sinus-air effect and (2) unreliable spectroscopy findings due to fat tissue in the orbital area and bone marrow in the skull base (Frodl et al., 2002). In addition, the statistical power of the MPFC analysis was not large due to the small sample size in each group.

The findings of the current study indicated that the cognitive function of the TRS patients was poorer than that of the non-TRS patients across several domains, especially in terms of the impact on WM (Arnsten et al., 2017; Bowie & Harvey, 2006; Fernandes et al., 2018). Several studies have attempted to examine the cognitive differences between TRS and non-TRS patients, using different measurements to assess cognitive function in the subgroups (McCutcheon et al., 2020; Thomas et al., 2017). Previous studies have indicated that dopaminergic control plays an important role in WM impairment and improvement before and after medical treatment (Arnsten et al., 2017; Tanaka, 2006). The ACC, as a part of the limbic system, modulates not only emotion, but also attention, which plays a pivotal role in WM (Lenartowicz & McIntosh, 2005; Luerding, Weigand, Bogdahn, & Schmidt-Wilcke, 2008; Otsuka, Osaka, Morishita, Kondo, & Osaka, 2006). The ACC mediates the linkage between the parietal lobe and the prefrontal cortex for central executive function (Chai, Abd Hamid, & Abdullah, 2018; Deserno et al., 2012; Kondo et al., 2004). The significant difference in the relationship of WM with the ACC glutamatergic level between the TRS and non-TRS patients in our study was in agreement with similar studies (Reddy-Thootkur et al., 2020); however, empirical studies of the correlation between WM and glutamatergic level in TRS and non-TRS patients are scarce.

Studies of HCs and patient groups have reported poorer cognitive function in patient groups, especially TRS groups, particularly with regards to WM, owing to tendencies toward an older age and a lower educational level (Chai et al., 2018; Kondo et al., 2004). In the current study, we confirmed that WM was related to the glutamatergic level in the ACC in the patients with non-TRS. This finding was similar to previous evidence indicating that acute or chronic stress affects intra- and extracellular glutamatergic concentrations in animal models (Yuen, Wei, & Yan, 2017) and the dopamine–WM relationship in humans (Cools & D'Esposito, 2011). An inverted U-shaped relationship was identified for the association between WM and connectivity of the dorsolateral prefrontal cortex (Callicott et al., 2000; Cools & D'Esposito, 2011; Lenartowicz & McIntosh, 2005; Perlstein et al., 2001), and we speculated that the same mechanisms could exist in the ACC. However, no associations were found between WM and glutamatergic levels in the HC and TRS groups in previous studies. Whether this inverted U-shaped relationship exists between glutamate and WM is unclear. Another potential model is an upside-down exponential function with concave down decreasing. For healthy subjects on the left-hand side of the curve, the glutamate level is normal, and does not harm WM, while for patients with non-TRS, the glutamate level tends toward being over the normal range, and WM function is affected in the pattern of a negative association. In patients with TRS, glutamate levels are extremely high, and WM is severely damaged. The association between glutamate and WM is weak.

We had proposed that (Huang et al., 2020) patients with TRS with a long-term full dopaminergic antagonist prognosis were characterized by a higher glutamatergic concentration in the brain. In this case, without efficacious treatment to relieve chronic stress, cognitive decline may be irreversible. Considering the hypothesis of glutamate in TRS and non-TRS, glutamatergic modulators such as sodium benzoate, sarcosine, or lamotrigine may represent new promising treatments to improve cognitive function in patients with schizophrenia, TRS, or other severe psychiatric disorders (Chang, Lin, Liu, Chen, & Lane, 2020; Lin et al., 2020a, 2020b). Moreover, the results are diverse based on different brain regions, age distributions, schizophrenia subgroups, and cognition measurements. In conclusion, cognitive dysfunction in different brain areas and classification of schizophrenia based on the glutamatergic hypothesis remain unclear, and are still plausible. Further research and studies are required for further investigation to inform future clinical practice.

The current study had several potential strengths. It is important to note that the TRS and non-TRS patients in the study were admitted to the chronic ward or half-way house of the same hospital, and were subject to the same food supplements, rehabilitation programs, and drug-monitoring systems. In addition, we employed the latest data in the analysis that indicated correlations between WMI and glutamatergic neurometabolites in the TRS patients, while no associations were observed in the non-TRS and HC groups.

We acknowledge some limitations of this study, which may have led to the unexplained results and are worthy of mention. The first was that the equipment used for MRI scanning at 1.5-Tesla disturbed detection of the signals of glutamate, which did not have adequate resolution to measure the signal of glutamine clearly. Therefore, we attempted to obtain the glutamatergic concentration ratio to NAAx rather than the pure glutamate concentration, as has been employed for analysis in some studies (Reddy-Thootkur et al., 2020). Second, we selected a higher dosage of antipsychotics (equivalent to more than 600 mg CPZ) to indicate TRS; hence, the TRS participants were classified based on a high clozapine dosage, and those on a low clozapine dosage were classified as non-TRS patients. The cognitive function of the participants in this study was measured using the WAIS-IV IQ, which is not commonly used for the evaluation of cognition in studies of schizophrenia patients; other studies have employed RBANS, the Wisconsin Card-Sorting Test, and the continuous performance test. The TRS patients tried their best to complete the IQ test with an experienced psychologist present, rather than being relied upon to obtain measurements by themselves, due to their lower cognitive function as compared with the non-TRS and HC groups. Although the study included 92 participants whose ACC regions were scanned correctly, for one-third of the participants in whom the same technician, time point, and procedures were employed, a poor quality index resulted, and hence these participants were excluded from the MPFC analysis. This may be one of the reasons for the fewer significant findings in the MPFC. Though moderated measurement criteria were used as modulated quality, the ROIs of the ACC and MPFC were measured manually for each individual, which led to non-uniformity. Not all subtypes of schizophrenia were examined in this study; in particular, first-episode schizophrenia patients and those at high risk of psychosis were not included in this research.

The drugs used in the two groups differed greatly. Although our findings remained robust after controlling for certain drugs (see online Supplementary Tables S7–S9), this should be validated in a further experimental study, in which drug use in the two groups was matched. Finally, the sample size of the HC group was small as compared with groups in other published studies on schizophrenia.

In summary, we found a higher ACC glutamatergic concentration in the TRS patients than that in the other two groups, and the glutamatergic neurometabolite levels in the ACC of the non-TRS patients were negatively associated with WM and were independent of covariates such as age and sex. Based on the glutamatergic hypothesis previously reported, an elevated glutamatergic distribution leads to increases in psychotic symptoms and decreases in cognitive function based on NMDA receptor or AMPA receptor dysfunction. In clinical practice and research, some glutamatergic modulators, such as sodium benzoate, valproic acid, lamotrigine, and sarcosine, have been efficacious in the treatment of schizophrenia and other psychiatric disorders (Li et al., 2019; Lin et al., 2020a, 2020b). Although the underlying mechanisms of the glutamatergic concentration in different cognitive function subgroups in TRS and non-TRS patients are still unknown, the glutamatergic concentration as estimated by MRS may be a personalized predictive biomarker or treatment response detector for patients with schizophrenia.

## Supplementary material

For supplementary material accompanying this paper visit https://doi.org/10.1017/S003329172100533X. click here to view supplementary material
