# Supplementary material for: Altered glutamate level and its association with working memory among patients with treatment-resistant schizophrenia (TRS): a proton magnetic resonance spectroscopy study
Source: Psychol Med. 2022 Feb 24;53(7):3220–7. doi: 10.1017/S003329172100533X (PMC10244010; doi:10.1017/S003329172100533X)
Supplement: Supplementary file 1 [file S003329172100533Xsup.zip › S003329172100533Xsup001.docx]

**Supplementary Table 1.** Cognitive function measurements of the study participants, stratified into three groups.

| Characteristic |  | Non-TRS (n = 35) |  | TRS (n = 38) |  | Control (n = 19) |  | Test statistic | | |
| --- | --- | --- | --- | --- | --- | --- | --- | --- | --- | --- |
|  |  | Mean (SD) |  | Mean (SD) |  | Mean (SD) |  | χ^2^/t/F | p | Post-hoc |
| Gender (male/female) |  | 17/18 |  | 21/17 |  | 9/10 |  | 0.47 | 0.8 |  |
| Cognitive function ratings |  |  |  |  |  |  |  |  |  |  |
| FSIQ |  | 81.91 (13.73) |  | 69.74 (16.03) |  | 107.26 (12.74) |  | 42.13 | < 0.01 | Control > Non-TRS > TRS |
| WMI |  | 84.49 (15.01) |  | 74.48 (14.23) |  | 101.63 (16.13) |  | 21.07 | < 0.01 | Control > Non-TRS > TRS |

Abbreviations: TRS, treatment-resistant schizophrenia; FSIQ, full-scale IQ; WMI, working memory index.

**Supplementary Table 2.** Medication categories classified by antipsychotic agents and mood stabilizers in the non-TRS and TRS patients.

| Characteristic |  | Non-TRS (n = 35) |  | TRS (n = 38) |  | Test statistic | | |
| --- | --- | --- | --- | --- | --- | --- | --- | --- |
|  |  | Mean (SD) |  | Mean (SD) |  | χ^2^/t/F | p | Post-hoc |
| Gender (male/female) |  | 17/18 |  | 21/17 |  | 0.47 | 0.80 |  |
| Smoking status |  | 16/19 |  | 13/25 |  | 1.007 | 0.316 |  |
| Dosage (CPZ equivalents) |  | 467.91 (186.48) |  | 821.36 (181.71) |  | 8.2 | < 0.01 |  |
| Clozapine (proportion) |  | 3/32 |  | 23/15 |  | 9.37 | < 0.02 |  |
| Clozapine (CPZ equivalent) |  | 17.14 (60.57) |  | 221.05 (280.60) |  | 4.21 | < 0.01 |  |
| Clozapine-like agent (proportion) |  | 5/30 |  | 12/26 |  | 3.05 | 0.08 |  |
| Clozapine-like agent (CPZ equivalent) |  | 55.00 (142.43) |  | 111.84 (269.03) |  | 1.55 | 0.13 |  |
| 1^st^ antipsychotic agent only (proportion) |  | 6/29 |  | 0/38 |  | 11.15 | < 0.01 |  |
| 2^nd^ antipsychotic agent only (proportion) |  | 18/17 |  | 7/31 |  | 8.82 | < 0.01 |  |
| 1^st^ and 2^nd^ ˇcombination (proportion) |  | 8/27 |  | 30/8 |  | 22.97 | < 0.01 |  |
| Mood stabilizers |  |  |  |  |  |  |  |  |
| Lithium |  | 2/33 |  | 6/32 |  | 1.90 | 0.17 |  |
| Anticonvulsant agent |  | 3/32 |  | 11/27 |  | 4.88 | < 0.01 |  |

Abbreviations: TRS, treatment-resistant schizophrenia; CPZ, chlorpromazine.

Note: clozapine-like agents: clothiapine, zotepine; 1^st^ antipsychotic agent: haloperidone, flupentixol, sulpiride; 2^nd^ antipsychotic agent: amisulpiride, aripiprazole, risperidone, lurasidone, quetiapine, olanzapine, paliperidone; anticonvulsant agent: phenytoin, carbamazepine, valproic acid, lamotrigine, clonazepam.

**Supplementary Table 3.** Analysis of covariance (ANCOVA) for testing of the group difference in Glx/NAAx level in the ACC, controlling for age and sex.

| Source | Sum of square | df | MS | F | p-value |
| --- | --- | --- | --- | --- | --- |
| Sex | 0.24 | 1 | 0.24 | 2.75 | 0.10 |
| Age | 0.16 | 1 | 0.16 | 1.76 | 0.19 |
| TRS | 0.91 | 2 | 0.46 | 5.17 | 0.008* |
| Error | 7.67 | 87 | 0.09 |  |  |
| Total | 8.77 | 91 |  |  |  |

Abbreviations: TRS, treatment-resistant schizophrenia; ACC, anterior cingulate cortex; Glx, total glutamate + glutamine; NAAx, N-acetylaspartate + N-acetylaspartylglutamate; MS: mean square.

*p < 0.05

**Supplementary Table 4.** Analysis of covariance (ANCOVA) for testing of the group difference in Glx/NAAx level in the ACC, controlling for age, sex, educational level, smoking status and cognitive function.

| Source | Sum of square | df | MS | F | p-value |
| --- | --- | --- | --- | --- | --- |
| Sex | 0.23 | 1 | 0.23 | 2.65 | 0.11 |
| Age | 0.18 | 1 | 0.18 | 2.01 | 0.16 |
| Education | 0.16 | 1 | 0.16 | 1.85 | 0.18 |
| Smoking status | 0.04 | 1 | 0.04 | 0.46 | 0.50 |
| FSIQ | 0.00 | 1 | 0.00 | 0.00 | 0.95 |
| TRS | 0.63 | 2 | 0.32 | 3.60 | 0.03* |
| Error | 7.39 | 84 | 0.09 |  |  |
| Total | 8.77 | 91 |  |  |  |

Abbreviations: TRS, treatment-resistant schizophrenia; ACC, anterior cingulate cortex; Glx, total glutamate + glutamine; NAAx, N-acetylaspartate + N-acetylaspartylglutamate; FSIQ, full-scale IQ; MS: mean square.

*p < 0.05

**Supplementary Table 5.** Analysis of covariance (ANCOVA) for testing of the group difference in Glx/NAAx level in the MPFC, controlling for age and sex.

| Source | Sum of square | df | MS | F | p-value |
| --- | --- | --- | --- | --- | --- |
| Sex | 0.08 | 1 | 0.08 | 0.23 | 0.63 |
| Age | 0.32 | 1 | 0.32 | 0.93 | 0.34 |
| TRS | 0.15 | 2 | 0.07 | 0.21 | 0.81 |
| Error | 24.27 | 70 | 0.35 |  |  |
| Total | 24.89 | 74 |  |  |  |

Abbreviations: TRS, treatment-resistant schizophrenia; MPFC, medial prefrontal cortex; Glx, total glutamate + glutamine; NAAx, N-acetylaspartate + N-acetylaspartylglutamate; FSIQ, full-scale IQ; MS: mean square.

*p < 0.05

**Supplementary Table 6.** Analysis of covariance (ANCOVA) for testing of the group difference in Glx/NAAx level in the MPFC, controlling for age, sex, educational level, smoking status and cognitive function.

| Source | Sum of square | df | MS | F | p-value |
| --- | --- | --- | --- | --- | --- |
| Sex | 0.06 | 1 | 0.06 | 0.16 | 0.69 |
| Age | 0.4 | 1 | 0.40 | 1.11 | 0.30 |
| Education | 0.13 | 1 | 0.13 | 0.35 | 0.56 |
| Smoking status | 0.00 | 1 | 0.00 | 0.00 | 0.99 |
| FSIQ | 0.033 | 1 | 0.033 | 0.09 | 0.77 |
| TRS | 0.14 | 2 | 0.07 | 0.19 | 0.82 |
| Error | 24.13 | 67 | 0.36 |  |  |
| Total | 24.89 | 74 |  |  |  |

Abbreviations: TRS, treatment-resistant schizophrenia; MPFC, medial prefrontal cortex; Glx, total glutamate + glutamine; NAAx, N-acetylaspartate + N-acetylaspartylglutamate; FSIQ, full-scale IQ; MS: mean square.

*p < 0.05

**Supplementary Table 7.** Analysis of covariance (ANCOVA) for testing of the group difference in Glx/NAAx level in the ACC, controlling for age, sex, lithium usage proportion and mood stabilizer proportion.

| Source | Sum of square | df | MS | F | p-value |
| --- | --- | --- | --- | --- | --- |
| Sex | 0.34 | 1 | 0.34 | 3.33 | 0.11 |
| Age | 0.34 | 1 | 0.34 | 3.41 | 0.16 |
| Lithium-P | 0.01 | 1 | 0.01 | 0.05 | 0.18 |
| Anticonvulsant agent-P | 0.01 | 1 | 0.01 | 0.07 | 0.5 |
| TRS | 0.94 | 1 | 0.94 | 9.34 | 0.003* |
| Error | 6.76 | 67 | 0.1 |  |  |
| Total | 8.11 | 72 |  |  |  |

Abbreviations: TRS, treatment-resistant schizophrenia; ACC, anterior cingulate cortex; Glx, total glutamate + glutamine; NAAx, N-acetylaspartate + N-acetylaspartylglutamate; MS: mean square.

Note: anticonvulsant agent: phenytoin, carbamazepine, valproic acid, lamotrigine, clonazepam.

*p < 0.05

**Supplementary Table 8.** Analysis of covariance (ANCOVA) for testing of the group difference in Glx/NAAx level in the ACC, controlling for age, sex, 1^st^ antipsychotic usage proportion, 2^nd^ antipsychotic proportion and combined antipsychotic proportion.

| Source | Sum of square | df | MS | F | p-value |
| --- | --- | --- | --- | --- | --- |
| Sex | 0.35 | 1 | 0.35 | 3.33 | 0.11 |
| Age | 0.33 | 1 | 0.33 | 3.41 | 0.16 |
| 1^st^ antipsychotic-P | 0.10 | 1 | 0.10 | 0.05 | 0.18 |
| 1^st^ antipsychotic-P | 0.04 | 1 | 0.04 | 0.07 | 0.5 |
| Combined antipsychotic-P | 0.06 | 1 | 0.06 | 0.64 | 0.43 |
| TRS | 0.74 | 1 | 0.74 | 7.39 | 0.008* |
| Error | 6.59 | 66 | 0.1 |  |  |
| Total | 8.11 | 72 |  |  |  |

Abbreviations: TRS, treatment-resistant schizophrenia; ACC, anterior cingulate cortex; Glx, total glutamate + glutamine; NAAx, N-acetylaspartate + N-acetylaspartylglutamate; MS: mean square.

Note: 1^st^ antipsychotic agent: haloperidole, flupentixol, sulpiride; 2^nd^ antipsychotic agent: amisulpiride, aripiprazole, risperidone, lurasidone, quetiapine, olanzapine, paliperidone.

*p < 0.05

**Supplementary Table 9.** Analysis of covariance (ANCOVA) for testing of the group difference in Glx/NAAx level in the ACC, controlling for age, sex, clozapine usage proportion and clozapine-like agent proportion.

| Source | Sum of square | df | MS | F | p-value |
| --- | --- | --- | --- | --- | --- |
| Sex | 0.35 | 1 | 0.35 | 3.57 | 0.07 |
| Age | 0.33 | 1 | 0.33 | 3.43 | 0.06 |
| Clozapine-P | 0.21 | 1 | 0.21 | 2.17 | 0.15 |
| Clozapine-like agent-P | 0.06 | 1 | 0.06 | 0.62 | 0.44 |
| TRS | 1.19 | 1 | 1.19 | 12.24 | 0.001* |
| Error | 6.54 | 66 | 0.1 |  |  |
| Total | 8.11 | 72 |  |  |  |

Abbreviations: TRS, treatment-resistant schizophrenia; ACC, anterior cingulate cortex; Glx, total glutamate + glutamine; NAAx, N-acetylaspartate + N-acetylaspartylglutamate; P: proportion; MS: mean square.

Note: clozapine-like agent: clothiapine, zotepine.

*p < 0.05
